# A mid-Cambrian tunicate and the deep origin of the ascidiacean body plan

**DOI:** 10.1038/s41467-023-39012-4

**Published:** 2023-07-06

**Authors:** Karma Nanglu, Rudy Lerosey-Aubril, James C. Weaver, Javier Ortega-Hernández

**Affiliations:** 1 Museum of Comparative Zoology and Department of Organismic and Evolutionary Biology, Harvard University, Cambridge, MA 02138 USA; 2 Wyss Institute for Biologically Inspired Engineering, Harvard University, Cambridge, MA 02138 USA

**Keywords:** Palaeontology, Zoology, Palaeoecology

## Abstract

Tunicates are an evolutionarily significant subphylum of marine chordates, with their phylogenetic position as the sister-group to Vertebrata making them key to unraveling our own deep time origin. Tunicates greatly vary with regards to morphology, ecology, and life cycle, but little is known about the early evolution of the group, e.g. whether their last common ancestor lived freely in the water column or attached to the seafloor. Additionally, tunicates have a poor fossil record, which includes only one taxon with preserved soft-tissues. Here we describe *Megasiphon thylakos* nov., a 500-million-year-old tunicate from the Marjum Formation of Utah, which features a barrel-shaped body with two long siphons and prominent longitudinal muscles. The ascidiacean-like body of this new species suggests two alternative hypotheses for early tunicate evolution. The most likely scenario posits *M. thylakos* belongs to stem-group Tunicata, suggesting that a biphasic life cycle, with a planktonic larva and a sessile epibenthic adult, is ancestral for this entire subphylum. Alternatively, a position within the crown-group indicates that the divergence between appendicularians and all other tunicates occurred 50 million years earlier than currently estimated based on molecular clocks. Ultimately, *M. thylakos* demonstrates that fundamental components of the modern tunicate body plan were already established shortly after the Cambrian Explosion.

## Abstract (web summary)

Our understanding of the origins of tunicates, an important group of filter-feeding marine invertebrate chordates, is limited due to a poor fossil record. Here, the authors present a 500 million year old tunicate fossil, demonstrating that the modern tunicate body plan was established shortly after the Cambrian Explosion.

## Introduction

**Fig. 1:**
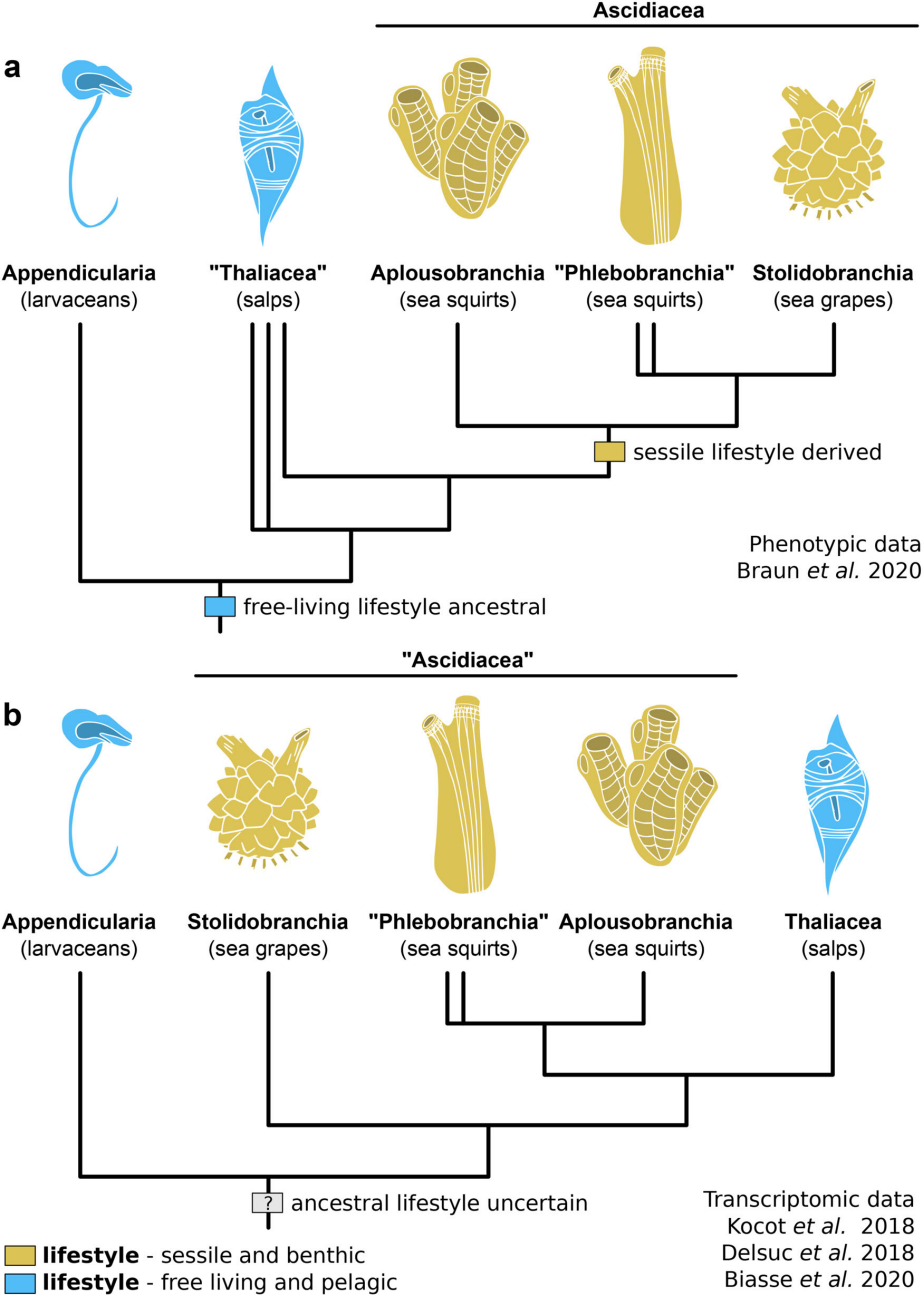
Competing hypotheses for phylogenetic relationships among extant tunicates. **a** Phenotypic data supports the monophyly of ascidiaceans, all of which are sessile and epibenthic as adults, and suggests the paraphyly of the free living and pelagic thaliaceans^5^. **b** Transcriptomic data suggests that ascidiaceans are paraphyletic, and supports monophyly of thaliaceans^4,6,8^.

The tunicates, or urochordates, include over 3000 extant species of marine invertebrates whose defining feature is the presence of a cellulosic extracellular matrix surrounding the body, which in most forms is known as the tunic^1^. Within Chordata, tunicates occupy a phylogenetic position as sister-group to vertebrates^2^, which makes them directly relevant for understanding the evolution of our subphylum. Tunicates are also characterized by enormous morphological and life history disparity that complicates understanding their origins in deep time^3^. Current morphological and molecular phylogenies of Tunicata support the presence of two major groups^4–6^. The first group consists of the appendicularians (ca. 75 species), pelagic and solitary animals superficially resembling tadpoles that filter pico- to nanoplankton using an excreted cellulose-rich “house”^1^. The second group comprises the ascidiaceans (Aplousobranchia, Phlebobranchia, and Stolidobranchia; ca. 2800–3000 species) and the thaliaceans (Doliolida, Pyrosomida, and Salpida; ca. 85 species)^4,6^. Ascidiaceans are species rich and diverse with regards to life history, morphology and ecology, including colonial and solitary epibenthic forms^5^, filter feeding and carnivorous representatives^7^, and a biphasic lifecycle where a pelagic tadpole-like larva metamorphoses into an epibenthic sessile adult^5^. By contrast, the thaliaceans are almost exclusively filter feeders and are characterized by a holopelagic life cycle that may be either monophasic (without larval period and metamorphosis) or biphasic^4,6^. Although still supported by a recent morphological phylogeny^5^ (Fig. 1a), the monophyly of ascidiaceans has been repeatedly challenged by molecular phylogenies, which recover thaliaceans nested within paraphyletic Ascidiacea as the sister group of phlebobranchs and aplousobranchs^4,6,8^ (Fig. 1b). Regardless of the interrelationships between ascidiaceans and thaliaceans, the appendicularians are consistently recovered as reciprocally monophyletic to the rest of the tunicates in both morphological and molecular phylogenies^4–6^. Thus, one of the most elusive questions in chordate evolution is whether the last common ancestor of Tunicata was a tadpole-like free living animal (as in appendicularians), or a sessile animal with paired siphons that lived attached to the benthos (as in ascidiaceans)^3,9^. Unfortunately, the fossil record has remained largely silent with regards to tunicate origins and macroevolution^8^. It consists of microscopic biomineralized spicules dating back to the Triassic period^10^, and possibly the macroscopic soft remains of the enigmatic *Shankouclava anningense* from the early Cambrian of South China^11^ (Supplementary Table 1). The overall appearance of *Shankouclava* resembles modern stalked ascidiaceans, but problematically lacks clear ascidiacean synapomorphies (e.g., paired siphons). Here we describe an exceptionally preserved tunicate macrofossil from the middle Cambrian Marjum Formation of Utah that illuminates the origins of the ascidiacean body plan and the early evolutionary history of the tunicates broadly.

## Results and discussion

### Systematic paleontology

Chordata (Linnaeus 1758)

Tunicata (Lamarck 1816)

*Megasiphon thylakos* gen. et sp. nov

#### Etymology

From Greek *Mega* (large) and *siphon* (siphon), referring to the prominent siphons. Species name from Greek *thylakos* (sac, pouch), refers to the sac-like body.

#### Diagnosis

**Fig. 2:**
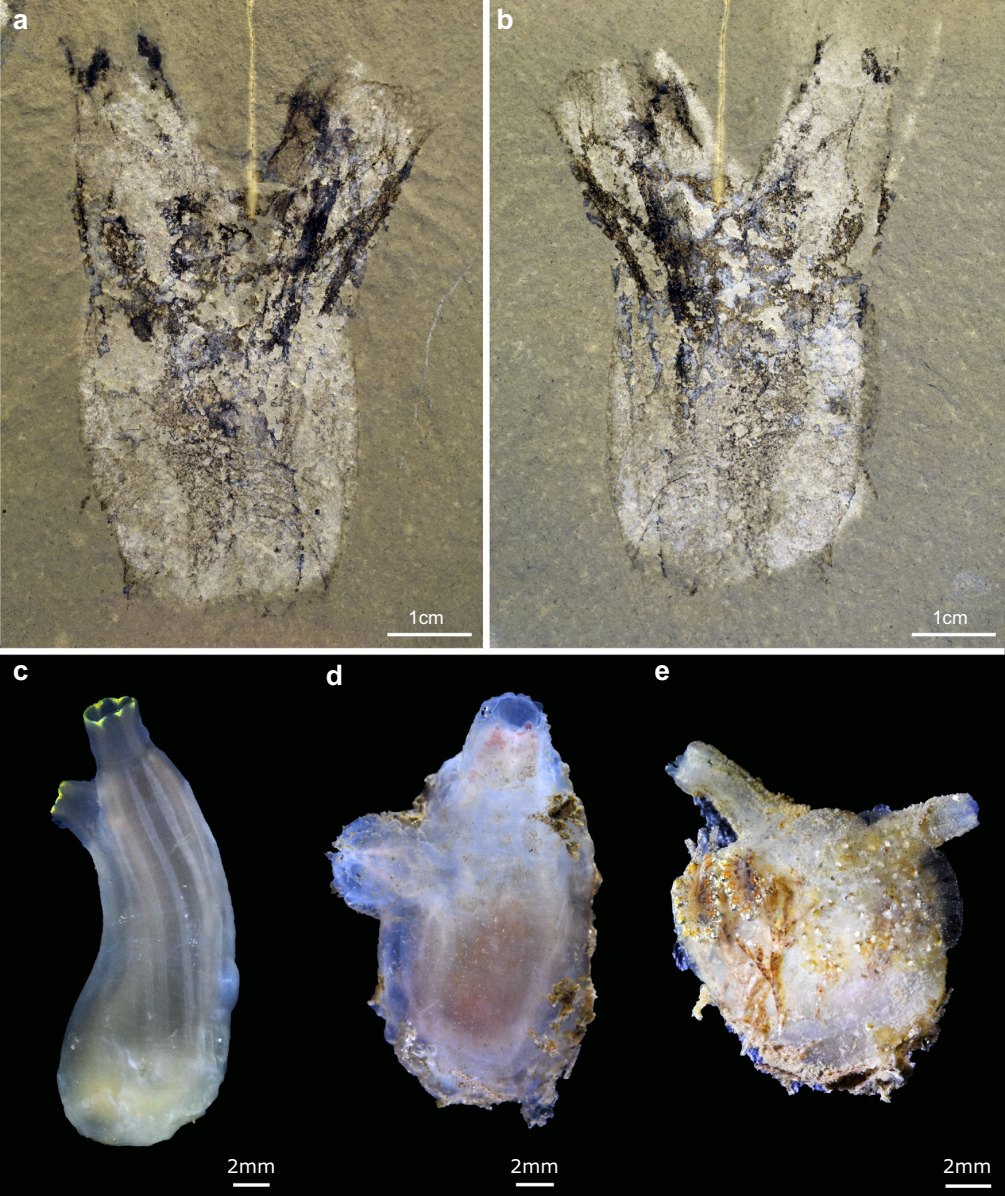
The tunicate *Megasiphon thylakos* nov. from the mid-Cambrian (Drumian) Marjum Formation of Utah and comparisons with modern benthic tunicates. **a** Holotype (UMNH.IP.6079) and only known specimen of *Megasiphon thylakos*, showing overall morphology including paired siphons and barrel-shaped body drawing immediate comparisons with modern benthic tunicates (**c**–**e**). **b** Counterpart to a. **c** *Ciona intestinalis* (Phlebobranchia). **d** *Ascidiella* sp. (Phlebobranchia) **e** *Molgula manhattensis* (Stolidobranchia).

Barrel-shaped main body extends apically into two similarly sized, long siphons (Fig. 2a, b). Main body with millimetric circular transverse muscle bands. Siphons project at roughly a 25° angle relative to longitudinal axis of main body, and are associated with longitudinal muscle bands extending from the upper region of the main body.

#### Locality and horizon

House Range, western Utah, USA; middle Marjum Formation, *Ptychagnostus punctuosus* Biozone^12^ (Drumian; Methods: Geological Setting).

### Description

**Fig. 3:**
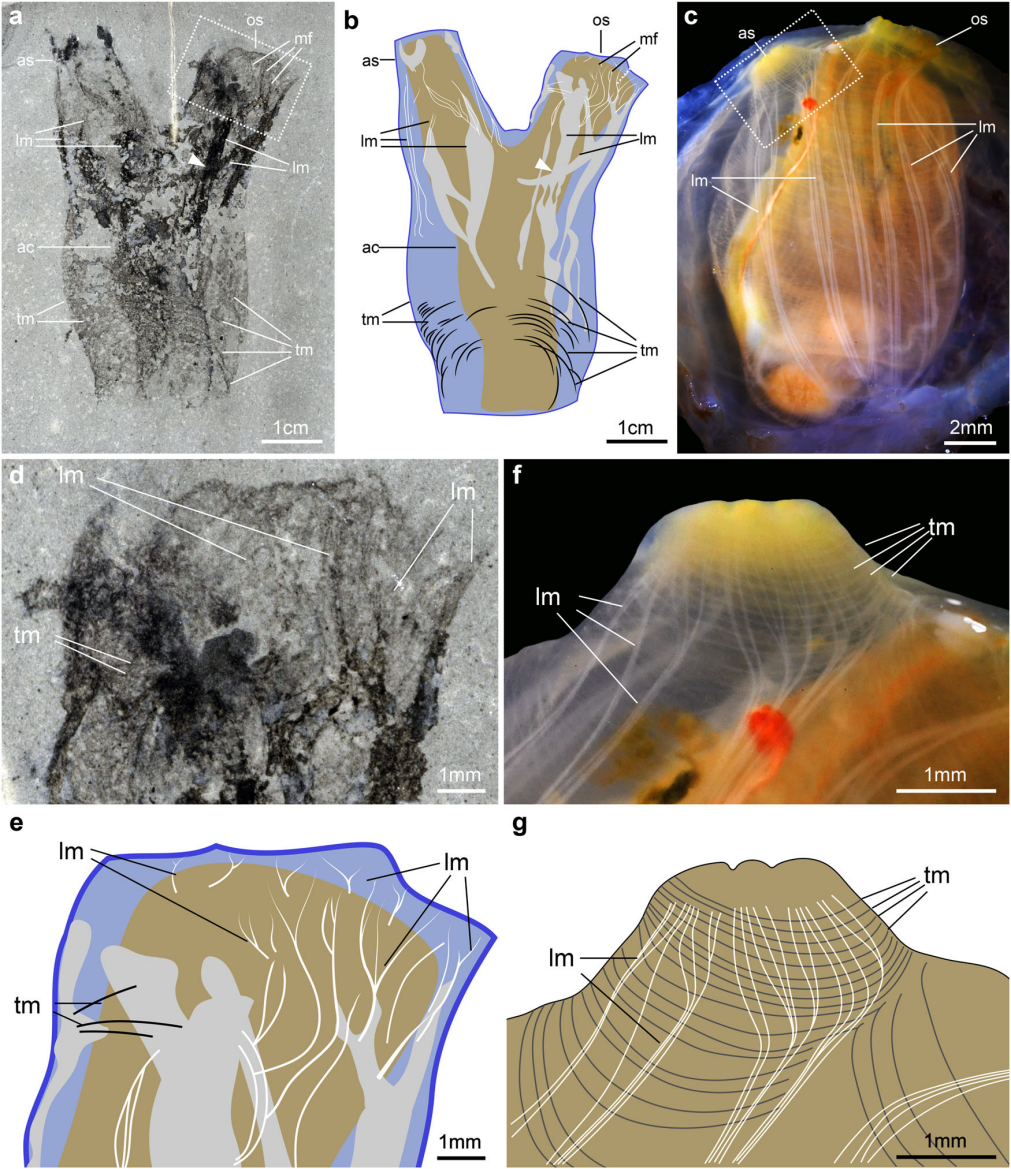
Anatomical details of *Megasiphon thylakos* nov. **a** The holotype (UMNH.IP.6079) preserves details of multiple internal anatomical structures, including prominent longitudinal muscle bands which diverge into thin, individual fibers as they enter the apical regions of the siphon. **b** Line drawing of (**a**). **c** Dissection of the modern ascidiacean *Ciona intestinalis*. The longitudinal muscles are easily visible, extending from the base of the animal (panel bottom) into both the oral and apical siphons. **d** Close up of boxed area in (**a**), showing the thin longitudinal muscle fibers. **e** Line drawing of (**d**). **f** Close up of boxed area in (**c**), showing the arrangement of muscles around the siphon. Note the pitchfork-like frayed arrangement of the longitudinal muscles around the siphon, highly reminiscent of the organization of *Megasiphon thylakos*. **g** Line drawing of (**f**). ac atrial cavity, as atrial siphon, lm longitudinal muscles, mf muscle fibers, os oral siphon, tm transverse muscles. White arrowheads in (**a**) and (**b**) indicate crossover points between adjacent longitudinal muscles.

The holotype and only known specimen (UMNH.IP.6079) of *Megasiphon thylakos* is preserved as a 3.2 cm long carbonaceous film on a mudstone matrix, and combines details of both the external and internal anatomy. The overall organization consists of a barrel-shaped main body and two similarly sized (0.8–0.9 cm long) apical outgrowths (Figs. 2a, b and 3a,b). The main body is 1.9 cm wide apically, and 1.2 cm wide at its base. This basal region is not as clearly delimitated from the surrounding matrix as the rest of the body and therefore, it is possible that the main body was taller than preserved in the sole available specimen. The apical outgrowths are approximately two-thirds of the length of the main body and project at a 25° angle relative to its longitudinal axis. The longest outgrowth tapers distally and it is not clear whether it is completely preserved; the second outgrowth is untapered along its length and clearly delimited from the matrix distally. These structures are interpreted as siphons based on comparisons with extant solitary ascidiaceans in both morphology and location (Figs. 2c–e and 3c; Supplementary Discussion). Internally, a faint and dark impression occupies approximately the central half of the main body, mirroring its outline, and extending into both siphons (Fig. 3a, b). We refer to the entire area as the atrial cavity.

The preservation of the specimen does not permit us to discriminate the tunic from the mantle, but elements of musculature of the body wall can be observed in the forms of dark longitudinal structures occurring in the apical region of the main body and the two siphons. Close to the siphon openings, they diverge into thin, thread-like fibers, producing a frayed appearance similar to that of a pitchfork (Fig. 3a–e). We interpret these structures as muscle bands, which split into unbundled muscle fibers in the apical regions of the siphons as seen in modern ascidiaceans (Fig. 3c, f, g and Supplementary Data 1)^13^. These longitudinal muscle bands vary in thickness from 0.6 mm to 1.2 mm, and intersect each other to produce a cross or diamond shape (Fig. 3a, b); individual muscle fibers proximal to the apical region of the siphons are roughly 50 μm thick (Fig. 3c–g). We hypothesize that the siphon with more extensive unbundled muscular fibers and thicker muscle bands may correspond to the oral siphon, such structures possibly facilitating intake of water for suspension feeding compared to the less muscular atrial siphon (Fig. 3d–g). However, we acknowledge that it is not possible to unequivocally differentiate between the two siphons based on the available material. The main body also features thin (ca. 0.4 mm) and apically curved striations, which become more visible in the lateral regions of its bottom half where they reach a density of roughly 1.5 per mm. These striations are interpreted as the transverse muscle bands found in the body wall of modern tunicates (Fig. 3b, c, f, g).

### Anatomy and ecology

We reconstruct *Megasiphon thylakos* as an ascidiacean-like tunicate with a relatively simple internal anatomy (Figs. 2 and 3). A large atrial cavity occupies the central region of the body, longitudinal muscle bands extend from the top of the main body through to the distal tips of the siphons, and thin transverse muscle bands wrap around the main body.

In modern ascidiacean tunicates, a number of structures are found in the area referred to herein as the atrial cavity. Most notable are the branchial basket, the atrial cavity proper, the stomach and intestines. The branchial basket in particular is enlarged to facilitate the filter feeding ecology of ascidiaceans, and would occupy a similar space as the structure identified in *Megasiphon*. However, we are unable to discern any of the diagnostic features of the branchial basket, namely the perforations in the collagenous wall called stigmata (or gill slits). While the preservation of collagenous gill bars of both hemichordates and chordates from the Cambrian is well documented, the relative thinness of the collagenous branchial basket may have resulted in a lower overall preservation potential. Similarly, no clear divisions are observable within this impression, such that the possible differentiation of intestines or the stomach cannot be made. All these structures might have been present in *Megasiphon*, and simply not preserved in the only available specimen.

Siphons greatly vary in size, morphology, and disposition in extant ascidiaceans. This is illustrated in Fig. 2 with three modern taxa showing different siphon arrangements (note also the shape and size differences): an apical oral siphon with a partially lateral atrial siphon (Fig. 2c); an apical oral siphon with a fully lateral atrial siphon (Fig. 2d); and atrial and oral siphons noticeably diverging from each other (Fig. 2e). Siphon condition can vary even more radically among the tunicates, from being on opposing ends along the central axis in thaliaceans, to atrial openings being shared among individuals in some colonial tunicates such as *Botryllus*. However, these examples are unlike the organization found in *Megasiphon*, which falls within the range of well-studied benthic tunicates such as the genera *Ciona* and *Molgula*. It is also worth noting that the siphons themselves can contract in response to stimuli, and thus their in-life position may have varied slightly. This appears particularly likely when considering the prominent muscle tissues preserved in this fossil.

The presumed longitudinal musculature of *M. thylakos* is most closely reminiscent to that of the modern ascidiacean *Ciona intestinalis*, where longitudinal muscles similarly run through the main body and the siphons, then split into individual muscle fibers near the siphons’ openings^13^ (Fig. 3). However, the longitudinal muscle bands of *C. intestinalis* are comparatively thinner and run from the base (body posterior end) to the siphons without intersecting (Fig. 3c, f). Circular or transverse muscle bands occur in most ascidiaceans^14^, and a similar arrangement of transverse muscles are particularly visible in phalloidin-stained *Molgula* juveniles^15^. In modern representatives, contraction of the circular and longitudinal musculature causes the animal to squirt or to cower, respectively. Their presence in *M. thylakos* indicates that these typical behavioural traits of tunicates had already evolved approximately 500 million years ago.

The overall morphology of *M. thylakos* is most similar to that of solitary and unstalked ascidiaceans, particularly the phlebobranchs, with two siphons projecting dorso-laterally (Fig. 2c, d). The orientation and size of its siphons resemble those of molgulids, which are unstalked stolidobranchs (Fig. 2e). By contrast, *M. thylakos* lacks any clear similarity to stalked stolidobranchs or the exclusively pelagic thaliaceans, and its morphology is incompatible with the tadpole-like appendicularians. The new species differs substantially from the early Cambrian *Shankouclava*^11^ in having a less elongate main body, no stalk, and critically, two well-developed siphons*. M. thylakos* also possesses distinctive longitudinal and transverse muscle bands, features as-yet unobserved in any of the nine described specimens^16^ of *Shankouclava*. Based on these comparisons, we reconstruct the autecology of *M. thylakos* as a sessile epibenthic suspension feeder, traits shared with most modern ascidiaceans.

### Evolutionary implications

**Fig. 4:**
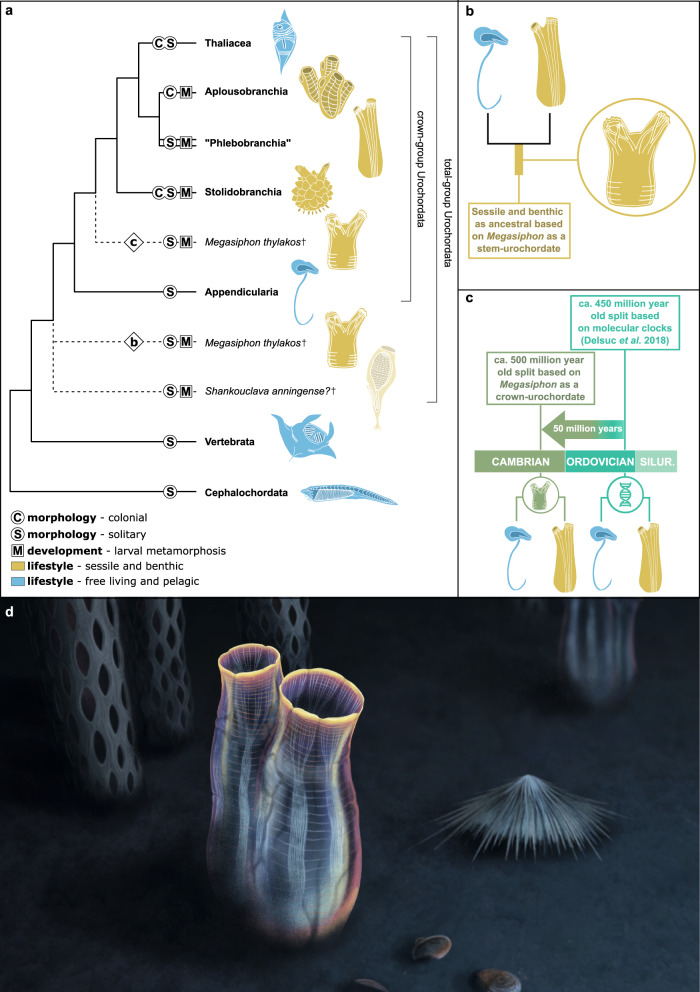
Evolutionary history of tunicates. **a** Simplified phylogeny of extant Tunicata follows ref. ^4^. Depending on the phylogenetic position of *Megasiphon thylakos*, a solitary, sessile, epibenthic organism with a biphasic life cycle that underwent larval metamorphosis is ancestral for either total-group Tunicata, or for non-appendicularian tunicates. **b** *M. thylakos* reconstructed as a stem-group tunicate would support a sessile mode of life in the adult forms as ancestral to Tunicata, as well as indirect development through a free-swimming larval form. **c** Simplified time scale of tunicate evolution. *M. thylakos* recovered as a crown-group tunicate would indicate that the ascidiacean body plan evolved during the mid-Cambrian (*Megasiphon* silhouette at the node in the diagram), approximately 50 million years earlier than the Late Ordovician dichotomy (double helix at the node in the diagram) estimated through molecular clocks^8^. **d** Artistic reconstruction of *M. thylakos*. Artwork by Franz Anthony.

The fundamentally ascidiacean-like body organization of *Megasiphon thylakos* supports its affinities within total-group Tunicata. Incorporating this new taxon into the morphological matrix of Braun et al. (2020) and analyzing this dataset using Bayesian inference invariably recover *M*. *thylakos* as a relatively derived ascidiacean tunicate (Supplementary Data 2 and Supplementary Code 1). However, relatively few characters can be confidently coded for *M. thylakos*, most of which are related to colonial versus non-colonial life histories, which is a highly variable characteristic within Tunicata^4^ (Supplementary Code 1). Further, it has been demonstrated that morphology alone cannot confidently recover many of the higher tunicate relationships which are now supported by large molecular datasets^4–6,8^. Given that external morphology alone may therefore be unable to precisely resolve the phylogenetic placement of this Cambrian taxon relative to extant tunicates, we argue that it is most conservative to propose two alternative and likely phylogenetic placements, which carry profound but significantly different evolutionary implications (Fig. 4a–c). As hypothesized for *Shankouclava*^11,16^, *M. thylakos* may represent a stem-group tunicate, a position that would indicate that an ascidiacean-like body plan and autecology are ancestral for total-group Tunicata, and the tadpole-like appendicularian ecomorph a derived state (Fig. 4b). Supported by developmental data^17^ and the report of massive gene loss in appendicularians^9^, this hypothesis would imply that the tunicate last common ancestor had a biphasic lifecycle, with larval metamorphosis and a solitary epibenthic adult. Alternatively, *M. thylakos* may occupy a more crownward position as the sister-group of the clade uniting ascidiaceans and thaliaceans (Fig. 4a). This placement would indicate that the divergence between appendicularians and all other tunicates had already occurred by the Drumian, pushing back this dichotomy by approximately 50 million years relative to the latest molecular clock estimates^8^ (Fig. 4c). This placement would further substantiate the emerging evolutionary pattern described in various phylogenetically distant marine invertebrates (e.g., demosponges^18^, magelonid polychaetes^19^, enteropneust^20,21^ and pterobranch hemichordates^22^, branchiopod crustaceans^23^) where the crown-group evolved shortly after, if not during the main pulse of the Cambrian Explosion. Under this evolutionary scenario, the precise morphology of the tunicate last common ancestor remains uncertain given the paucity of information within stem-group Tunicata. Based on the morphology of *M. thylakos* (Fig. 2) and molecular phylogenies of extant representatives, the earliest non-appendicularian tunicates were most likely epibenthic and solitary ascidiacean-like organisms with a biphasic life cycle that included larval metamorphosis. The uniphasic and pelagic lifestyle would then be synapomorphic for thaliaceans.

While neither hypothesis can be definitively rejected, we posit that the phylogenetic position of *M. thylakos* as a stem-group tunicate, rather than a stem-group ascidiacean, is the more likely scenario for two reasons. First, the phylogenetic placement for *M. thylakos* as a stem-group tunicate does not require assuming a substantial shift in origination date for Ascidiacea based on current estimates^8^. Instead, it would only place the minimum divergence estimates for the origins of Tunicata at ~500 mya (the age of the Marjum Formation), only 16 million years older than current molecular clock estimates (484-411 million years)^8^. In this context, we believe it would be reasonable to use this age for dating future trees, as the unusual morphology of *Shankouclava* makes its relationship to the rest of the tunicates somewhat ambiguous (i.e., unclear oral siphon and entirely lacking atrial siphon). Second, an evolutionary scenario implying an ascidiacean-like body plan as ancestral to the tunicates as a whole appears to be the generally best supported scenario when considering other lines of evidence.

*M. thylakos* also carries broader ramifications for our understanding of the Cambrian Explosion as a key interval for early animal diversification. Tunicates are notoriously absent during this time period, with only *Shankouclava* (which differs substantially from modern tunicates) having been described in 2003^11^, and no further convincing tunicate macrofossils having been discovered in the intervening two decades (Supplementary Table 1). Thanks to *M. thylakos*, tunicates join the roster of the major animal groups that had already achieved a markedly crown group-like morphology near the origins of metazoan phyla (Fig. 4d), but also begs the question: if morphologically modern tunicates were already well established by the middle Cambrian, where is their fossil record? Exceptional fossil deposits such as the Burgess Shale and Chengjiang preserve not just the most delicate taxa (e.g. annelids^24^, ctenophores^25^, chordates^26^), but also extraordinarily labile features such as nervous systems^27,28^ and guts with last meals intact^29,30^. The fact that the anatomically robust macroscopic tunicates appear to have been preferentially lost due to taphonomic factors such as pre-fossilization decay^31,32^ is highly perplexing.

Several possible scenarios may explain this absence. Tunicates may have become established alongside other major animal groups during the Cambrian Explosion, but then had a relatively low level of diversity throughout most of the Palaeozoic, precluding their chances of becoming well represented in the fossil record. They may also have existed at low abundances for most of their history, with the filter feeding dominance of early sponges and brachiopods occupying their modern ecological niche. Finally, despite their cosmopolitan distribution in modern oceans, early tunicates may have had more restricted biogeographic ranges or inhabited specific aquatic environments that might not be already represented by the major Cambrian sites with exceptional preservation. This last possibility highlights the pitfalls of extrapolating global, phylum-level patterns from a small subset of Cambrian localities, even ones as exceptional as the Burgess Shale or Chengjiang. The increasingly frequent discovery of new localities such as Marble Canyon^33^, Qingjiang^34^ and Haiyan^35^ are routinely redefining our picture of early metazoan communities and our understanding of the early stages of animal diversification. In this context, the lesser studied Cambrian Lagerstätten of Utah—namely the Spence Shale^36^, Wheeler Formation^37^, Weeks Formation^38^, and Marjum Formation^12^ from which *M. thylakos* originates—represent potential wellsprings of critical evolutionary, ecological, and taphonomic insights into the dynamics of the Cambrian world and the early diversification of animals.

## Methods

Ethical approval was not required for any of the specimens included in this study. Access to the fossil specimen of *Megasiphon thylakos* was permitted through the Natural History Museum of Utah in Salt Lake City, where the specimen is deposited (UMNH.IP.6079). Live tunicates were commercially purchased from the Marine Biological Laboratory, University of Chicago. Live specimens were euthanized with 50:50 mixture of magnesium chloride dissolved in the seawater that the tunicates were transported in, manually dissected for digital photography, and fixed in 70% ethanol. Specimens were photographed with a Nikon D7500 DSLR camera fitted with a Macro Nikkor 40 mm lens. Fossils were photographed underwater with cross-polarizing filters. All measurements were made in ImageJ, all figures were produced in Adobe Photoshop CS and Inskcape. The Bayesian phylogenetic analysis was conducted using MrBayes 3.2.7a and the following search parameters: 10 million generations, sampling every 1000 generations, 25% burn-in, using an mkv model with a gamma distribution for rate variation.

### Geological setting

The holotype (UMNH.IP.6079) of *Megasiphon thylakos* was collected in the middle part (*Ptychagnostus punctuosus* agnostoid Biozone^12^) of the Marjum Formation in the House Range of western Utah, USA. These mudstone-dominated strata have yielded a diverse middle Cambrian (Miaolingian: Drumian) fossil biota (ca. 100 species^12^). The Marjum Formation forms with the underlying Wheeler and overlying Weeks Formations – which both yield soft-bodied fossils too – a ca. 610 m-thick continuous succession of thin-bedded limestone and shale/marl^39^. These deep-water sediments were deposited in a fault-controlled trough within the offshore margin of a carbonate platform, which fringed the tropical northern margin of the palaeocontinent Laurentia (now western North America). The Marjum exceptional fauna is dominated by deposit feeding and predatory/scavenging euarthropods, but also comprises approximately 20 benthic filter feeding species including brachiopods, graptolites, and sponges^40^.

### Notes on character codings in Supplementary Code 1

Below is additional commentary on the coding decisions made in our character matrix (Supplementary Code 1) which is modified from Braun et al. 2020^5^. The vast majority of characters could not be scored, which is not unusual for fossil taxa, particularly those of this age. All characters that were not scored as “?” are listed below.

1 Sessile adults: (0) absent; (1) present. *Megasiphon* is scored as 1, as it was a sessile ascidiacean-like tunicate.

2 Undulatory locomotion in adults: (0) absent; (1) present. *Megasiphon* is scored as -, as this is an inapplicable character for taxa with sessile adults.

3 Body division: (0) absent; (1) present. *Megasiphon* is coded as absent, as no clear body divisions can be observed.

5 Incurrent and excurrent siphons on opposite poles of the animal: (0) absent; (1) present. *Megasiphon* is coded as present, as both siphons are prominent and easily recognizable.

12 Conspicuous and discrete circular muscle bands for locomotion: (0) absent; (1) present. *Megasiphon* is coded as absent, as this is a character describes the unique muscular arrangement found in doliolids and salps, which is not present in *Megasiphon*.

13 Shape of circular muscle bands: (0) discontinuous; (1) continuous. *Megasiphon* is coded as -, as this is a character that is dependent on character 12.

16 Calcareous spicules in tunic: (0) absent; (1) present. *Megasiphon* is coded as 0, as there is no evidence of spicules (which have been known to fossilize in younger strata).

24 Notochord in adults: (0) absent; (1) present. *Megasiphon* is coded as 0, as there is no evidence of a notochord.

25 Length of notochord: (0) not extending to the anterior end of the body; (1) extending along the entire body to its most anterior tip. *Megasiphon* is coded as -, as this is dependent on character 24 being present.

26 Coloniality: (0) absent; (1) present. *Megasiphon* is coded as 0, as it is not a colonial taxon.

27 Sexually mature colonial form: (0) absent: sexually propagating chain of animals break up; (1) present: sexual forms remain entirely colonial. *Megasiphon* is coded as – as this is dependent on character 26 being present.

28: Connection of zooids within colonies: (0) zooids completely embedded in a common tunic; (1) zooids connected via stolons. *Megasiphon* is coded as – as this is dependent on character 26 being present.

31 Type of budding: (0) epicardial; (1) mesenchymatic; (2) palleal; (3) complex stolo prolifer. *Megasiphon* is coded as -, as there is no evidence for budding.

32 Type of epicardial budding: (0) strobilation; (1) pyloric (esophageal and entero-epicardial). *Megasiphon* is coded as -, as there is no evidence for budding.

33 Type of strobilation: (0) abdominal; (1) postabdominal. *Megasiphon* is coded as -, as there is no evidence for budding.

34 Type of mesenchymatic budding: (0) septal; (1) vascular. *Megasiphon* is coded as -, as there is no evidence for budding.

57 Distinct excretory structure: (0) absent; (1) present. *Megasiphon* is coded as 0, as there is no evidence of a discrete excretory structure, as is the case in most tunicates.

58 Type of excretory structure: (0) renal sac (synonym: kidney; Van Name, 1945; Kott, 1985); (1) excretory vesicles or nephrocytes. *Megasiphon* is coded as -, as this is dependent on character 57 being present.

66 Position of rectum in species with a mostly posterior gastrointestinal tracts (character 64: 0): (0) dorsomedian; (1) ventromedian. *Megasiphon* is coded as 0, as there is no rectum but rather an excurrent siphon.

67 Atrium: (0) absent; (1) present. *Megasiphon* is coded as 1, as we are able to define an atrial cavity.

93 Adult cerebral eye(s): (0) absent; (1) present. *Megasiphon* is coded as 0, as it does not have eyes (like other tunicates).

94 Type of photoreceptor cells in adult cerebral eyes: (0) rhabdomeric; (1) ciliary. *Megasiphon* is coded as -, as this is dependent on character 93 being present.

95 Lens in adult cerebral eye: (0) absent; (1) present. *Megasiphon* is coded as -, as this is dependent on character 93 being present.

## Supplementary information

Supplementary Information

Description of Additional Supplementary Files

Supplementary Data 1

Supplementary Data 2

Supplementary Code 1

## Data Availability

All data are available within the supplementary information of this paper. This published work and the nomenclatural acts it contains have been registered in ZooBank, the proposed online registration system for the International Code of Zoological Nomenclature (ICZN). The ZooBank LSIDs (Life Science Identifiers) can be resolved and the associated information viewed through any standard web browser by appending the LSID to the prefix “http://zoobank.org/”. The LSIDs for this publication are: 588EC6D0-00CD-4D26-A0D5-118CDD402ECC.

## Code availability

The character matrix for the phylogenetic analysis can be found in Supplementary Code 1. No new code was generated for this paper.
