# Supplementary material for: A mid-Cambrian tunicate and the deep origin of the ascidiacean body plan
Source: Nat Commun. 2023 Jul 6;14:3832. doi: 10.1038/s41467-023-39012-4 (PMC10325964; doi:10.1038/s41467-023-39012-4)
Supplement: Supplementary file 1 — Supplementary Information [file 41467_2023_39012_MOESM1_ESM.pdf]

SUPPLEMENTARY INFORMATION: A mid-Cambrian tunicate and the deep origin of the ascidiacean body plan

Taxon	General description	Age	Reference
Ausia fenestrata	Internal cast of a pliable, sac-shaped organism, with perforations that may be similar to the pharyngeal basket of tunicates.	Vendian	Fedonkin et al. 2012
Burykhia hunti	Internal cast of a pliable, sac-shaped organism, with perforations that may be similar to the pharyngeal basket of tunicates.	Vendian	Fedonkin et al. 2012
Shankouclava anningense	Sac-like body with a stalk and single, presumably oral siphon. Perforated branchial basket; possible atrial pore and endostyle.	Cambrian (Stage 3)	Chen et al. 2003
Megasiphon thylakos nov.	Sac-like, unstalked body with paired siphons and a large atrial cavity. Prominent longitudinal muscles, thinner transverse muscles.	Cambrian (Drumian)	Current study
Khmeria spp.	Cone-shaped or club-like, with growth lines, an operculum, and a calcareous exoskeleton.	Triassic	Wendt 2018
Zardinosoma spp.	Club-like or globose, with multiple opercula and a calcareous exoskeleton.	Triassic	Wendt 2018
Didemnid spicules	Eight morphotypes of spicules belonging to the tunicate family Didemnidae, roughly spherical with most having spikes.	Triassic (Carnian) to Quaternary (Pleistocene)	Varol & Houghton, 1996; Łukowiak et al. 2016 and references therein

Supplemental table 1. Index of published fossil tunicates. Although tunicates have been reported in the literature, their fossils are extremely rare. Most putative fossil tunicates are either incompletely preserved (e.g. isolated biomineralized spicules[1–3]), have anatomies that are at odds with some aspects of tunicate morphology (e.g. *Shankouclava*[4]) or are problematic and unlikely to represent tunicates at all (e.g. *Ausia* and *Burykhia*[5]). As a result, these fossils have not contributed substantively to discussions regarding

tunicate evolution. Once considered a primitive tunicate[6], the Silurian taxon *Ainiktozoon* is not listed herein, since it has been convincingly reinterpreted as an arthropod[7].

2. Supplementary Discussion

Alternative affinities for *Megasiphon thylakos*. Based on its age, morphology, and locality of discovery, three possible alternative identities for *Megasiphon thylakos* besides tunicate bear consideration, but all three can be confidently dismissed. The first two are a branching, organic tube or a branching burrow produced by a presumably vermiform animal. For the former, no other fossil tubes from the Cambrian are similar in size and morphology to *M. thylakos*, as demonstrated by Robison and Conway Morris's review[8] of the enigmatic and vermiform taxa from the Cambrian of Utah. The lone fossil that may be comparable is *Margaretia dorus*, which does branch at relatively similar angles as the siphons of *Megasiphon*, and can be of comparable width. However, it is morphologically utterly unlike *Margaretia*, lacking its characteristic series of spirally arranged pores and fibrous construction[9]. Additionally, due to its variety of well-preserved internal anatomical features preserved as carbonaceous films (e.g. musculature), *M. thylakos* is incompatible with identification as a trace fossil, particularly when compared with similarly-sized putative traces from Cambrian deposits[10]. A branching alga remains the last possible alternative, however, there is little which could tie *M. thylakos* to this group. It lacks any clear morphological features characterizing well-known Cambrian taxa interpreted as algae (e.g., holdfasts with branching systems, filamentous texture, sheet or fan-like thallus construction; see[11], table 1), including those subsequently reinterpreted as animal dwellings[9,10,12].

References

1. Varol, O. & Houghton, S. D. A review and classification of fossil didemnid ascidian spicules. *J. Micropalaeontology* **15**, 135–149 (1996).
2. Łukowiak, M., Dumitriu, S. D. & Ionesi, V. First fossil record of early Sarmatian didemnid ascidian spicules (Tunicata) from Moldova. *Geobios* **49**, 201–209 (2016).

3. Wendt, J. The first tunicate with a calcareous exoskeleton (Upper Triassic, northern Italy). *Palaeontology* **61**, 575–595 (2018).

4. Chen, J. Y. *et al.* The first tunicate from the early Cambrian of South China. *Proc. Natl. Acad. Sci. U. S. A.* **100**, 8314–8318 (2003).

5. Fedonkin, M. A., Vickers-Rich, P., Swalla, B. J., Trusler, P. & Hall, M. A new metazoan from the Vendian of the White Sea, Russia, with possible affinities to the ascidians. *Paleontol. J.* **46**, 1–11 (2012).

6. Scourfield, D. J. An anomalous fossil organism, possibly a new type of chordate, from the Upper Silurian of Lesmahagow, Lanarkshire – Ainiktozoon loganense, gen. et sp. Nov. *Proc. R. Soc. B* **121**, 533–547 (1937).

7. Van der Brugghen, W., Schram, F. R. & Martill, D. M. The fossil Ainiktozoon is an arthropod. *Nature* **385**, 589–590 (1997).

8. Conway Morris, S. & Robison, R. A. More soft-bodied animals and algae from the Middle Cambrian of Utah and British Columbia. *Univ. Kansas Paleontol. Contrib.* (1988).

9. Nanglu, K., Caron, J.-B., Conway Morris, S. & Cameron, C. B. Cambrian suspension-feeding tubicolous hemichordates. *BMC Biol.* **14**, 9 (2016).

10. Nanglu, K. & Caron, J. Symbiosis in the Cambrian: enteropneust tubes from the Burgess Shale co-inhabited by commensal polychaetes. *Proc. R. Soc. B Biol. Sci.* (2021).

11. Bykova, N. *et al.* Seaweeds through time: Morphological and ecological analysis of Proterozoic and early Paleozoic benthic macroalgae. *Precambrian Res.* **350**, 105875 (2020).

12. LoDuca, S. T., Caron, J.-B., Schiffbauer, J. D., Xiao, S. & Kramer, A. A reexamination of Yuknessia from the Cambrian of British Columbia and Utah. *J. Paleontol.* **89**, 82–95 (2015).
