# Supplementary material for: A mid-Cambrian tunicate and the deep origin of the ascidiacean body plan
Source: Nat Commun. 2023 Jul 6;14:3832. doi: 10.1038/s41467-023-39012-4 (PMC10325964; doi:10.1038/s41467-023-39012-4)
Supplement: Supplementary file 2 — Description of Additional Supplementary Files [file 41467_2023_39012_MOESM2_ESM.pdf]

Description of Additional Supplementary Files

File Name: Supplementary Data 1
Description: Close up images of the longitudinal muscle bands of Megasiphon thylakos, indicated by white arrowheads.

File Name: Supplementary Data 2
Description: Bayesian phylogenetic tree including Megasiphon thylakos, placing it in a polytomy with the stolidobranchs and the majority of a paraphyletic "phlebobranch" clade. Refer to Methods for details of the analytical parameters.

File Name: Supplementary Code 1
Description: Character matrix used in the phylogenetic analysis of Megasiphon thylakos, adapted from Braun et al. 2020.
