# Supplementary figures and images for: A mid-Cambrian tunicate and the deep origin of the ascidiacean body plan
Source: Nat Commun. 2023 Jul 6;14:3832. doi: 10.1038/s41467-023-39012-4 (PMC10325964; doi:10.1038/s41467-023-39012-4)

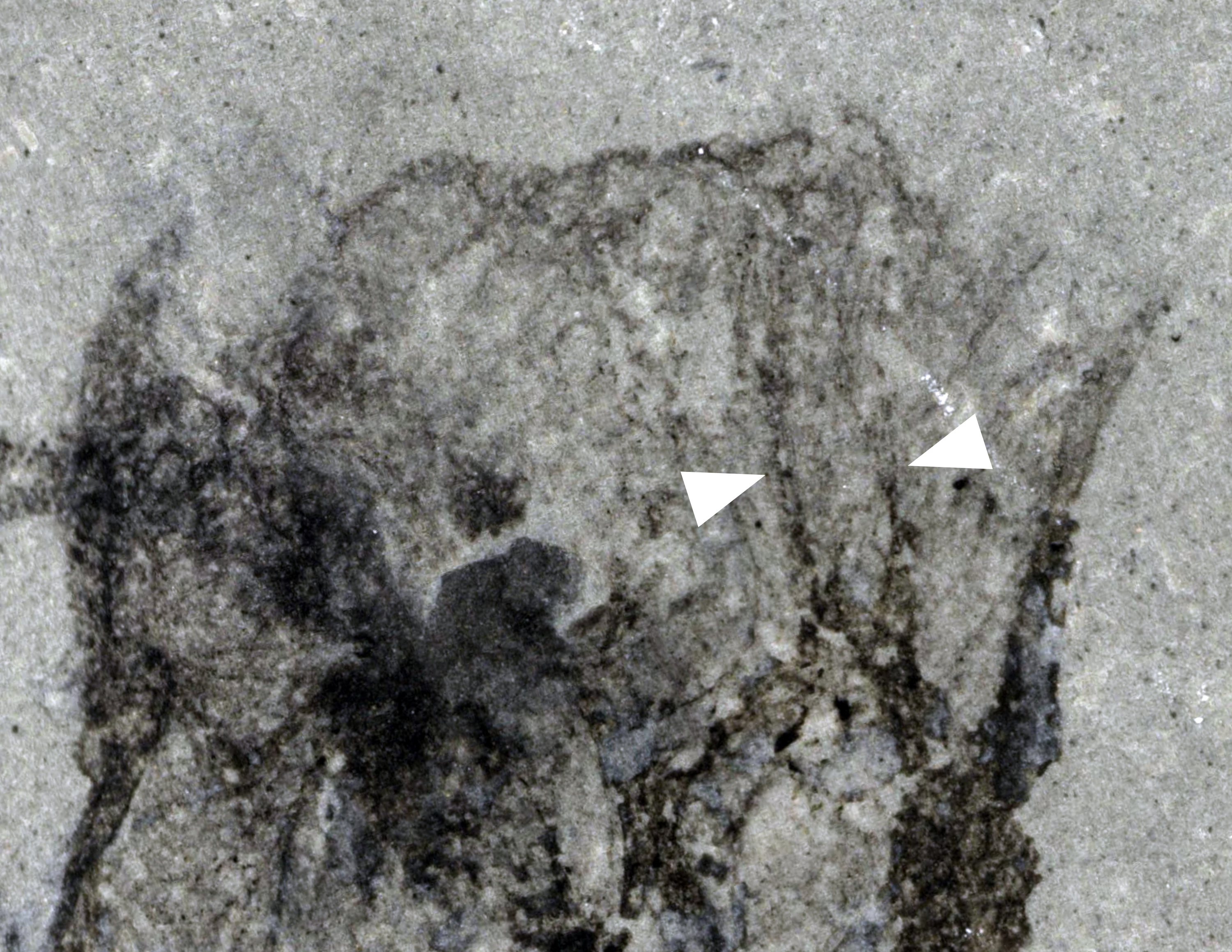

Supplement: Supplementary file 3 — Supplementary Data 1 [file 41467_2023_39012_MOESM3_ESM.png]

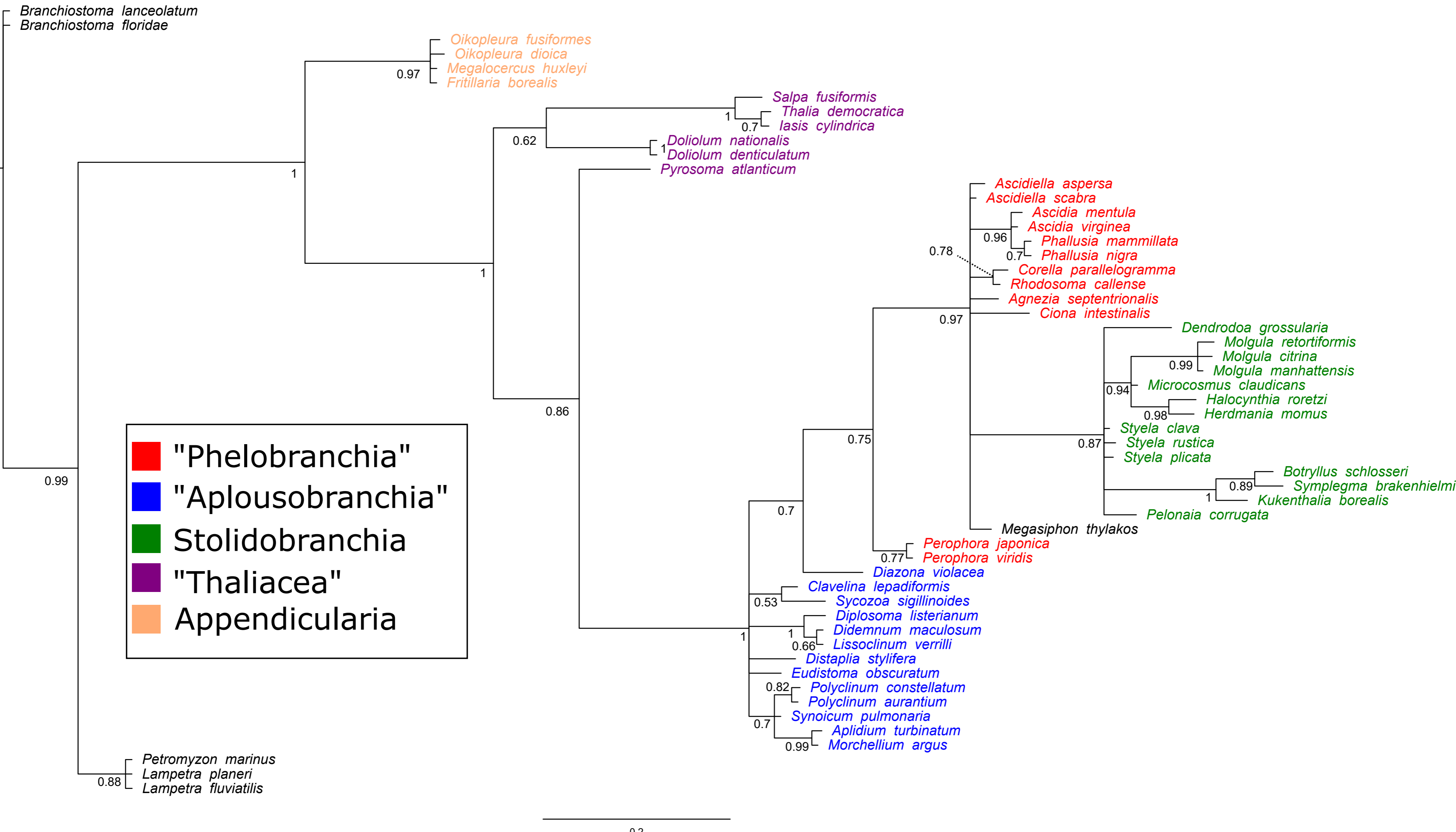

Supplement: Supplementary file 4 — Supplementary Data 2 [file 41467_2023_39012_MOESM4_ESM.pdf]
